# Crystal structure of clofarabine (form I), C_10_H_11_ClFN_5_O_3_, from synchrotron power diffraction data and density functional theory calculations

**DOI:** 10.1107/S2056989026006584

**Published:** 2026-06-26

**Authors:** Jacob K. Salazar, James A. Kaduk, Anja Dosen, Thomas N. Blanton

**Affiliations:** a North Central College, Department of Chemistry 131 S Loomis St Naperville IL 60540 USA; b North Central College, Department of Physics 131 S Loomis St Naperville IL 60540 USA; c Department of Chemistry, Illinois Institute of Technology, 3101 S. Dearborn St., Chicago IL 60616, USA; d ICDD, 12 Campus Blvd., Newtown Square, PA 19073-3273, USA; e ICDD, 12 Campus Blvd., Newtown Square, PA 19073-327,3 , USA; University of Aberdeen, United Kingdom

**Keywords:** powder diffraction, clofarabine, Clolar, Rietveld refinement, density functional theory

## Abstract

The crystal structure of clofarabine (form I) [systematic name: 2-chloro-9-(2-de­oxy-2-fluoro-β-d-arabino­furanos­yl)-9*H*-purin-6-amine], C_10_H_11_ClFN_5_O_3_, has been solved and refined using synchrotron X-ray powder diffraction data, and optimized using density functional theory techniques. The oxolane ring adopts an envelope conformation and the angle between the mean ring planes is 88.4 (2)°, resulting in an l-shaped mol­ecule. The mol­ecules stack along the short *a*-axis direction, and N—H⋯O, O—H⋯N and N—H⋯N hydrogen bonds link them into a three-dimensional network.

## Abstract 2

The crystal structure of clofarabine (form I) has been solved and refined using synchrotron X-ray powder diffraction data, and optimized using density functional theory techniques.

## 1. Chemical context

Clofarabine, C_10_H_11_ClFN_5_O_3_ (marketed as Clolar, Evoltra and Clofarex in different countries) is a purine nucleoside anti­metabolite, used to treat relapsed or refractory acute lymphoblastic leukemia in children and young adults (Bonate *et al.*, 2006 ▸). Clofarabine is administered intra­venously and functions by inhibiting DNA synthesis and ribonucleoside reductase. The systematic name (CAS Registry Number 123318-82-1) is (2*R*,3*R*,4*S*,5*R*)-5-(6-amino-2-chloro­purin-9-yl)-4-fluoro-2-(hy­droxy­meth­yl)oxolan-3-ol.
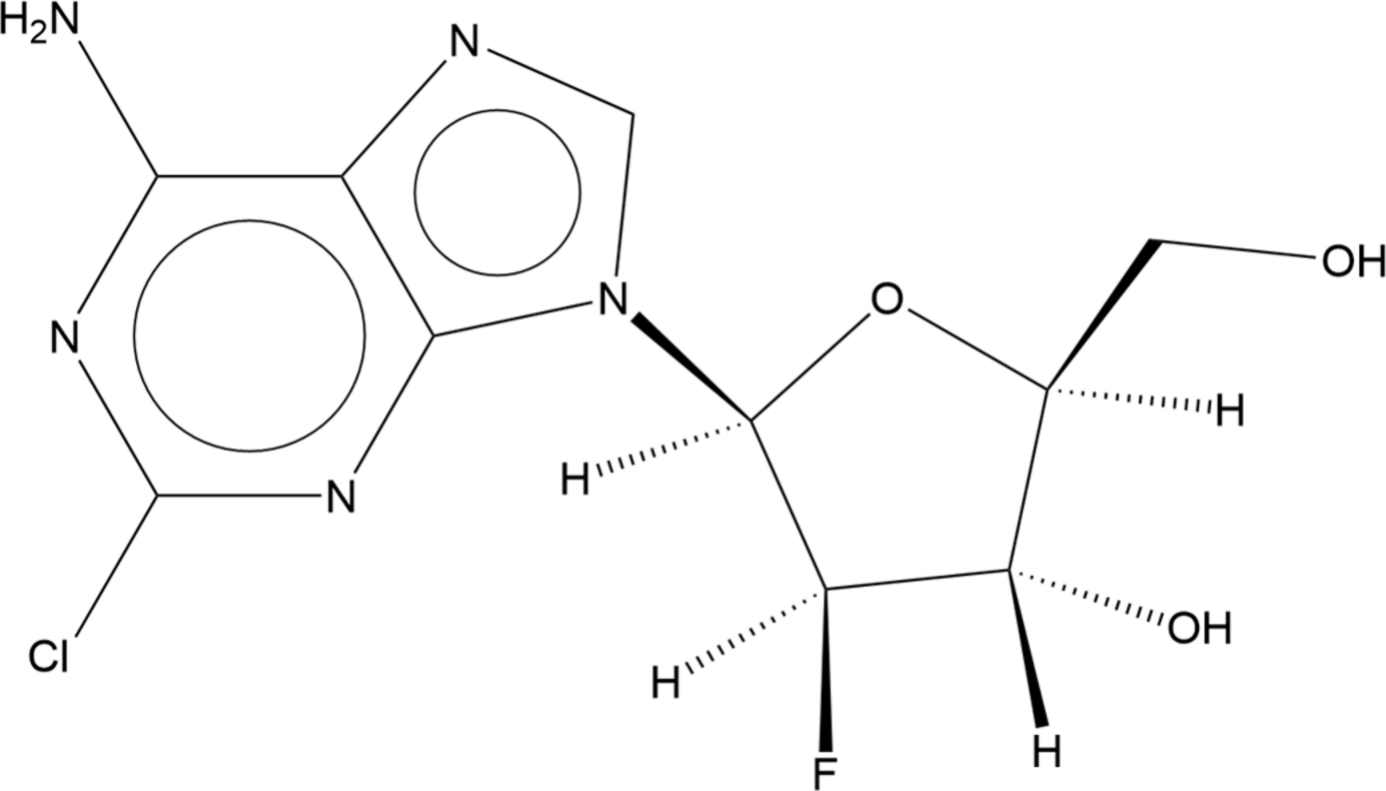


A powder pattern for clofarabine (form I) has been reported in Chinese Patent CN101407640A (Xia *et al.*, 2011 ▸). Un-named crystalline forms of clofarabine are claimed in US Patent 5,034,518 (Montgomery & Secrist, 1991 ▸; Southern Research Institute) and US Patent 5,661,136 (Montgomery & Secrist, 1997 ▸; Southern Research Institute). Xia *et al.* suggest that these earlier forms were monohydrates, and claim that their form is new. The present work was carried out as part of a project (Kaduk *et al.*, 2014 ▸) to determine the crystal structures of large-volume commercial pharmaceuticals, and includes high-quality powder diffraction data for them in the Powder Diffraction File (Kabekkodu *et al.*, 2024 ▸).

## 2. Structural commentary

**Figure 1:**
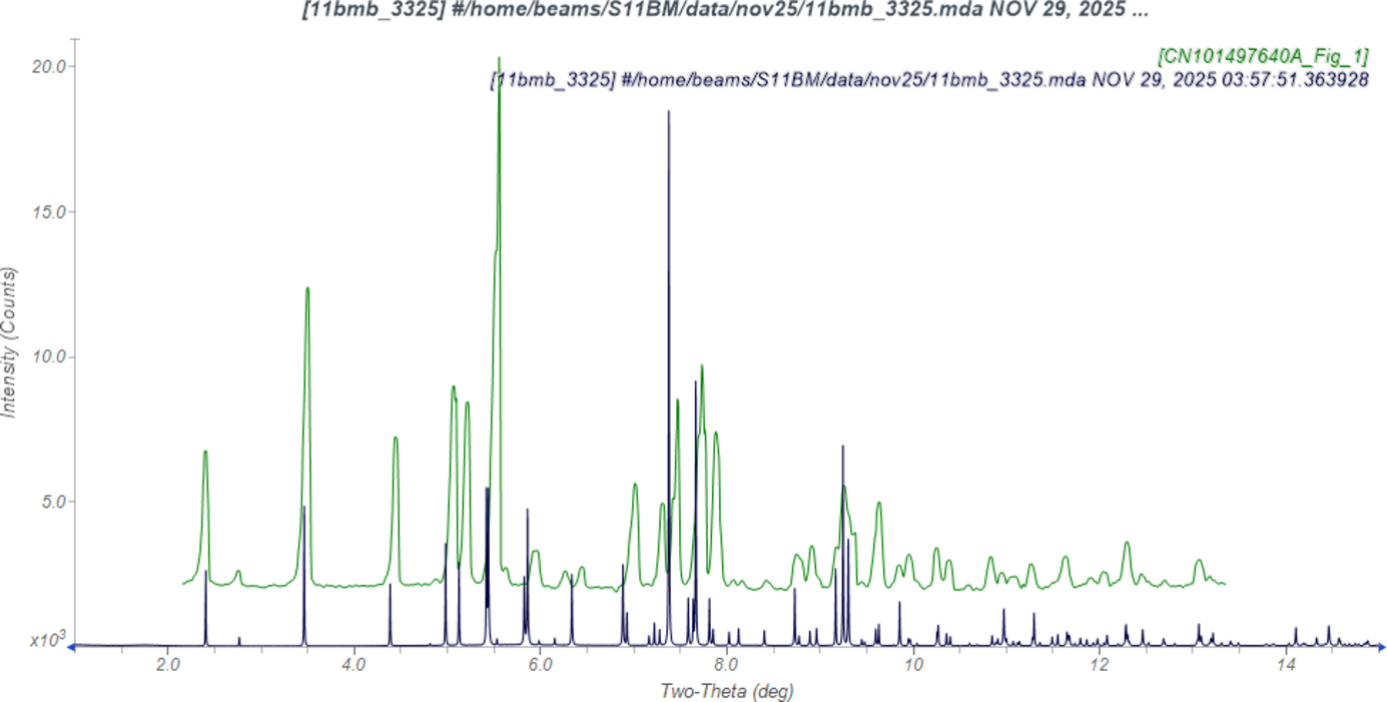
Comparison of the synchrotron pattern of clofarabine Form I (black) to that reported by Xia *et al.* (2011 ▸) using Cu *K*α radiation (green) converted to the synchrotron wavelength of 0.4687342 Å. The patent pattern (measured using Cu *K*α radiation) was digitized using *UN-SCAN-IT* (Silk Scientific, 2013 ▸) and converted to the synchrotron wavelength of 0.4687342 Å using *JADE Pro* (MDI, 2025 ▸). Image generated using *JADE Pro* (MDI, 2025 ▸).

The synchrotron X-ray powder pattern of clofarabine is similar enough to that reported by Xia *et al.* (2011 ▸) for form I (Fig. 1 ▸) to conclude that they represent the same material. The patent pattern exhibits significant displacement/transparency peak position errors, as well as substantial preferred orientation.

**Figure 2:**
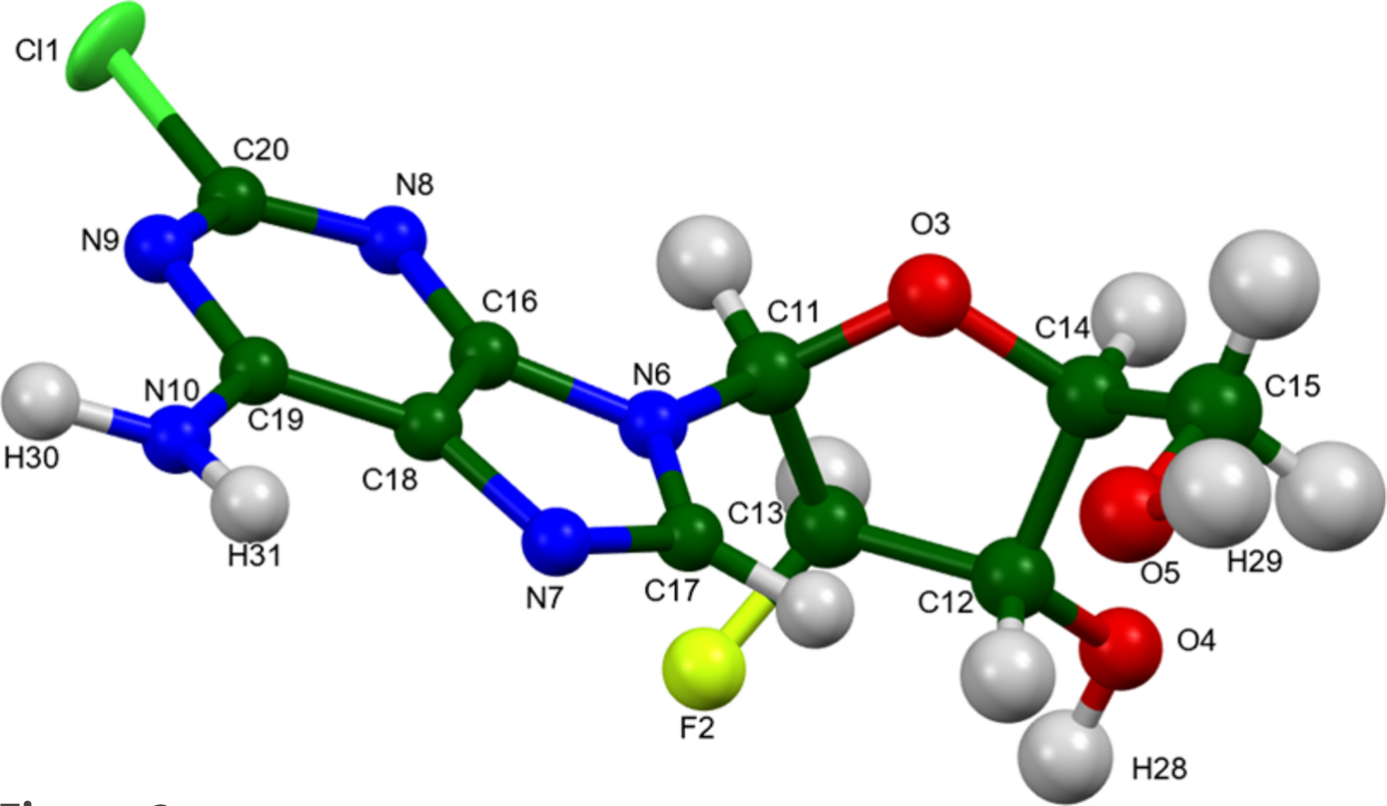
The mol­ecular structure of clofarabine, showing 50% probability spheroids/ellipsoids.

**Figure 3:**
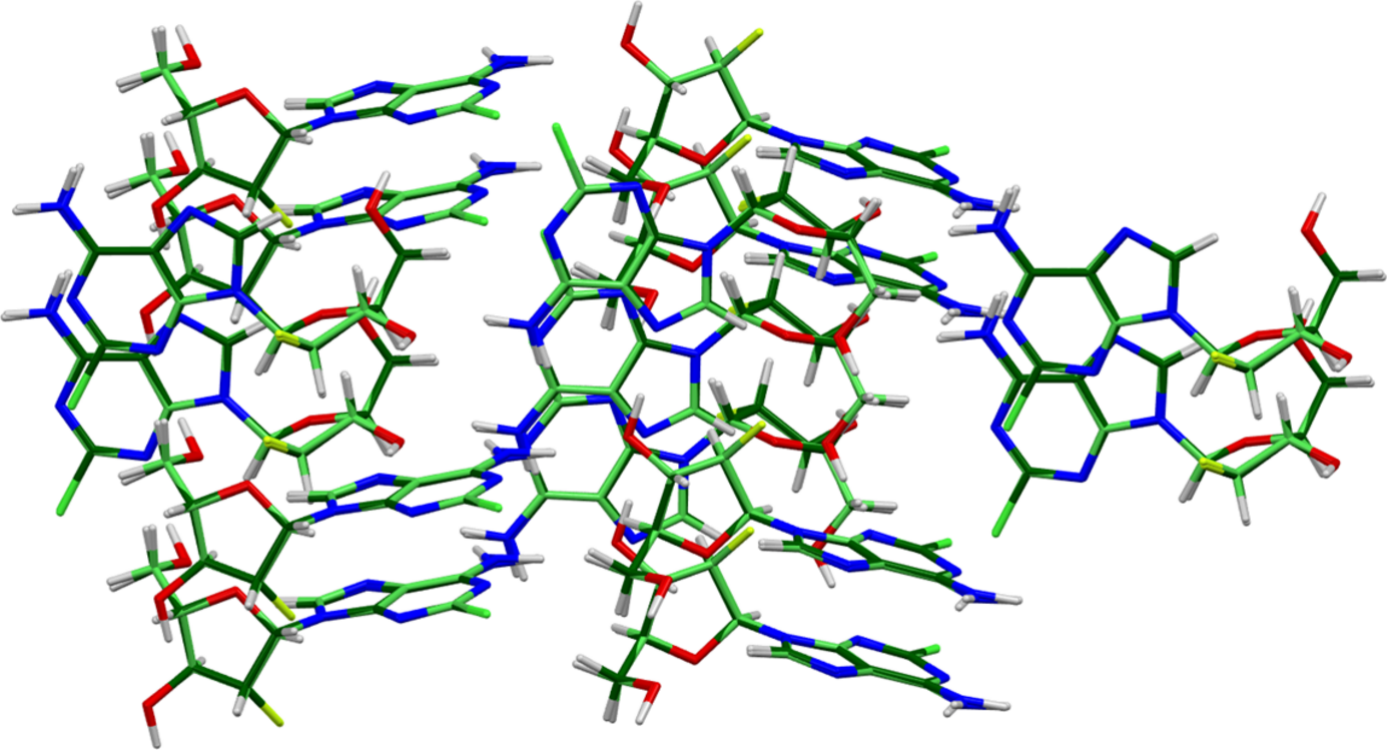
Comparison of the Rietveld-refined (colored by atom type) and *VASP*-optimized (pale green) structures of clofarabine, calculated using the *Mercury* CSD-Materials/Search/Crystal Packing Similarity tool. The root-mean-square Cartesian displacement is 0.078 Å.

**Figure 4:**
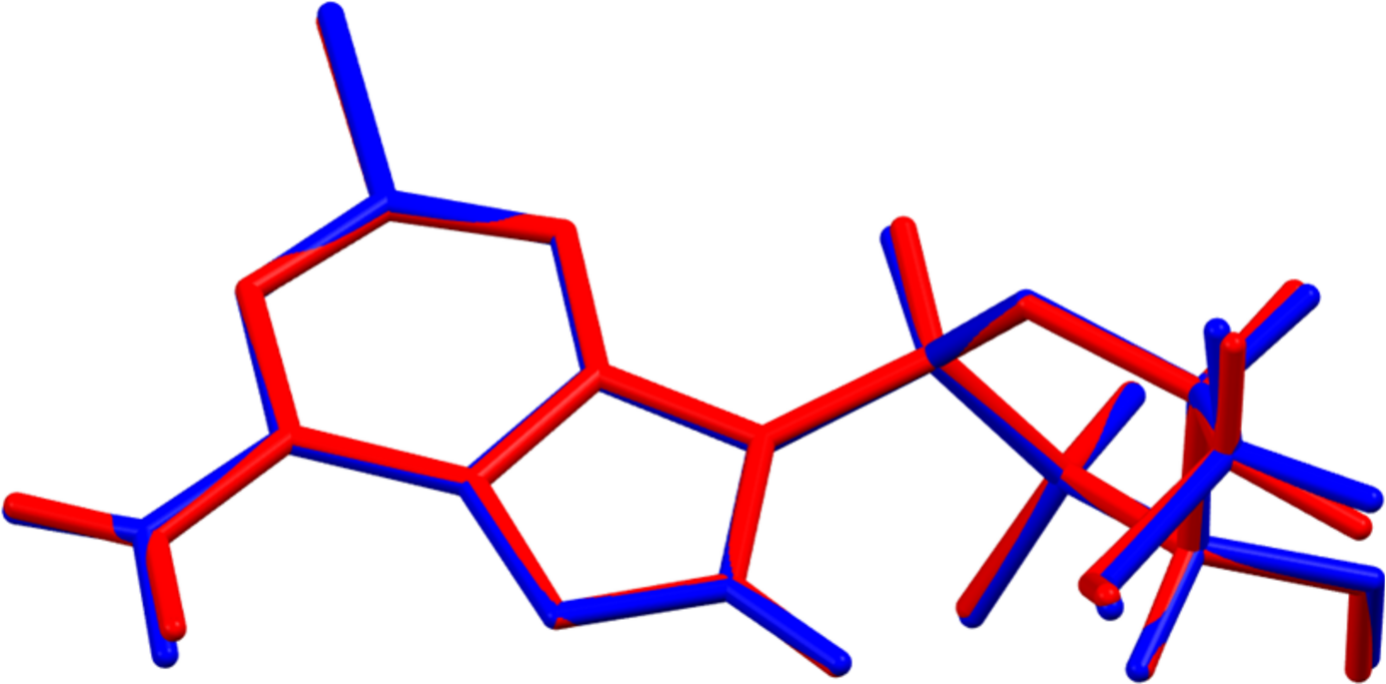
Comparison of the refined structure of clofarabine (red) to the *VASP*-optimized structure (blue). The comparison was generated using the Mercury Calculate/Mol­ecule Overlay tool; the r.m.s. difference is 0.048 Å.

The mean plane of the C16–C20/N6–N9 purine ring system of the asymmetric mol­ecule lies approximately in the (12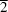) Miller plane, and the mean plane of the C11–C14/O3 oxolane ring is aligned approximately with (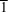
22). The latter ring adopts an envelope conformation with atom C12 as the flap. The angle between the mean ring planes is 88.4 (2)°, so the mol­ecules may be described as L-shaped (Fig. 2 ▸). The root-mean-square difference of the non-H atoms in the Rietveld-refined and *VASP*-optimized structures of clofarabine, calculated using the *Mercury* (Macrae *et al.*, 2020 ▸) CSD-Materials/search/crystal packing similarity tool is 0.078 Å (Fig. 3 ▸); the structures are essentially identical. The root-mean-square Cartesian displacement of the non-H atoms in the refined and optimized structures, calculated using the *Mercury* Calculate mol­ecule overlay tool, is 0.048 Å (Fig. 4 ▸). The agreements are within the normal range for correct structures (van de Streek & Neumann, 2014 ▸). The remaining discussion will emphasize the *VASP*-optimized structure.

All of the bond distances, bond angles, and torsion angles fall within the normal ranges indicated by a *Mercury* Mogul geometry check (Macrae *et al.*, 2020 ▸). Quantum chemical geometry optimization of the isolated clofarabine mol­ecule (DFT/B3LYP/6-31G*/water) using *Spartan ’24* (Wavefunction, 2025 ▸) indicated that the observed conformation is 5.2 kcal mol^−1^ higher in energy than a local minimum, which has a very similar conformation. The global minimum-energy conformation is 14.8 kcal mol^−1^ lower in energy, but is folded on itself to form intra­molecular O—H⋯N hydrogen bonds. Inter­molecular inter­actions are thus important in determining the observed solid-state conformation.

## 3. Supra­molecular features

**Figure 5:**
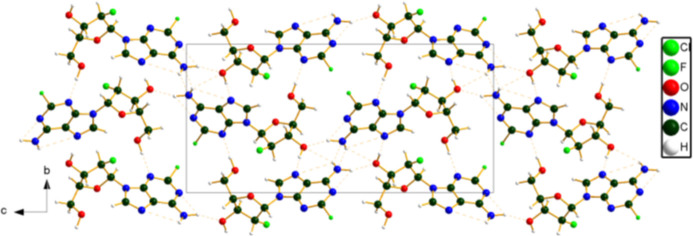
Crystal structure of clofarabine, viewed down the *a* axis.

**Figure 6:**
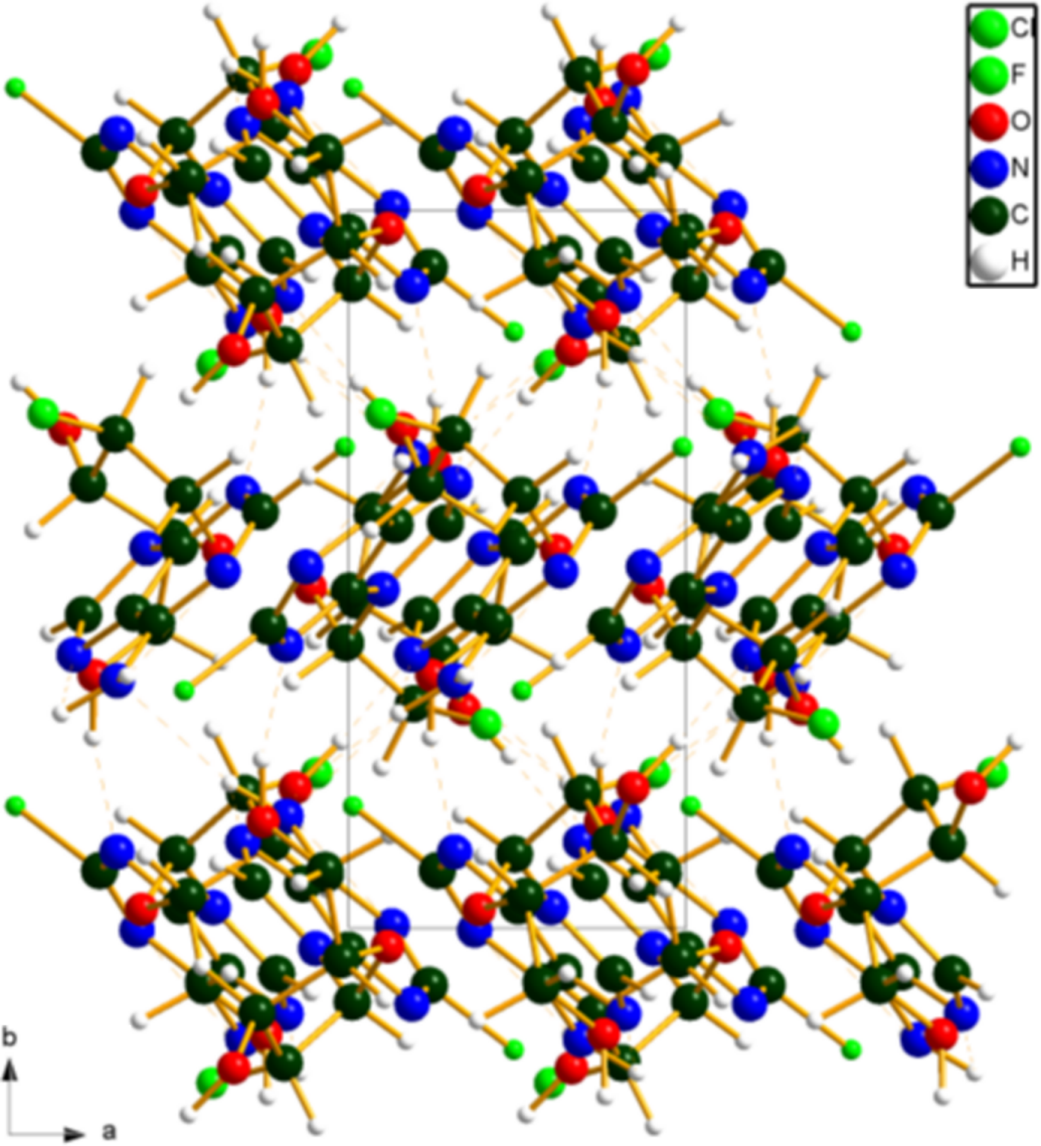
Crystal structure of clofarabine, viewed down the *c* axis.

Viewed down the short *a*-axis direction (Fig. 5 ▸) the structure exhibits discrete clofarabine mol­ecules. When viewed down the *c*-axis direction (Fig. 6 ▸) a herringbone arrangement of mol­ecules is apparent. The shortest ring centroid–ring centroid distance is 5.067 (2) Å, as the mol­ecules stack along the *a*-axis direction.

Analysis of the contributions to the total crystal energy of the structure using the Forcite module of *Materials Studio* (Dassault Systèmes, 2025 ▸) indicated that the intra­molecular energy is dominated by angle distortion terms, as might be expected for a mol­ecule containing a fused ring system. The inter­molecular energy is dominated by van der Waals attractions, which in this force-field based analysis include hydrogen bonds. The hydrogen bonds are better discussed using the results of the DFT calculation.

There are several hydrogen bonds in the structure (Table 1 ▸). The amino group N10 acts as a donor in two classical hydrogen bonds, one to the hydroxyl group O4 and to another amino group N10. The energy of the N10—H30⋯O4 hydrogen bond is 5.6 kcal mol^−1^, calculated using the correlation of Wheatley & Kaduk (2019 ▸). The hydroxyl groups O4 and O5 form strong O—H⋯N hydrogen bonds to the ring N atoms N7 and N8, respectively. These link the mol­ecules into chains along the *b*-axis direction, with graph set descriptors (Etter, 1990 ▸; Bernstein *et al.*, 1995 ▸; Motherwell *et al.*, 2000 ▸) 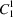
(8), 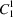
(9) and 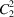
(11). The N—H⋯O and N—H⋯N hydrogen bonds link the mol­ecules along the *c*-axis direction, with graph sets 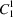
(10) and 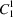
(2). These and other larger patterns result in a three-dimensional hydrogen bond network. Four intra- and inter-mol­ecular C—H⋯O hydrogen bonds also contribute to the lattice energy.

**Figure 7:**
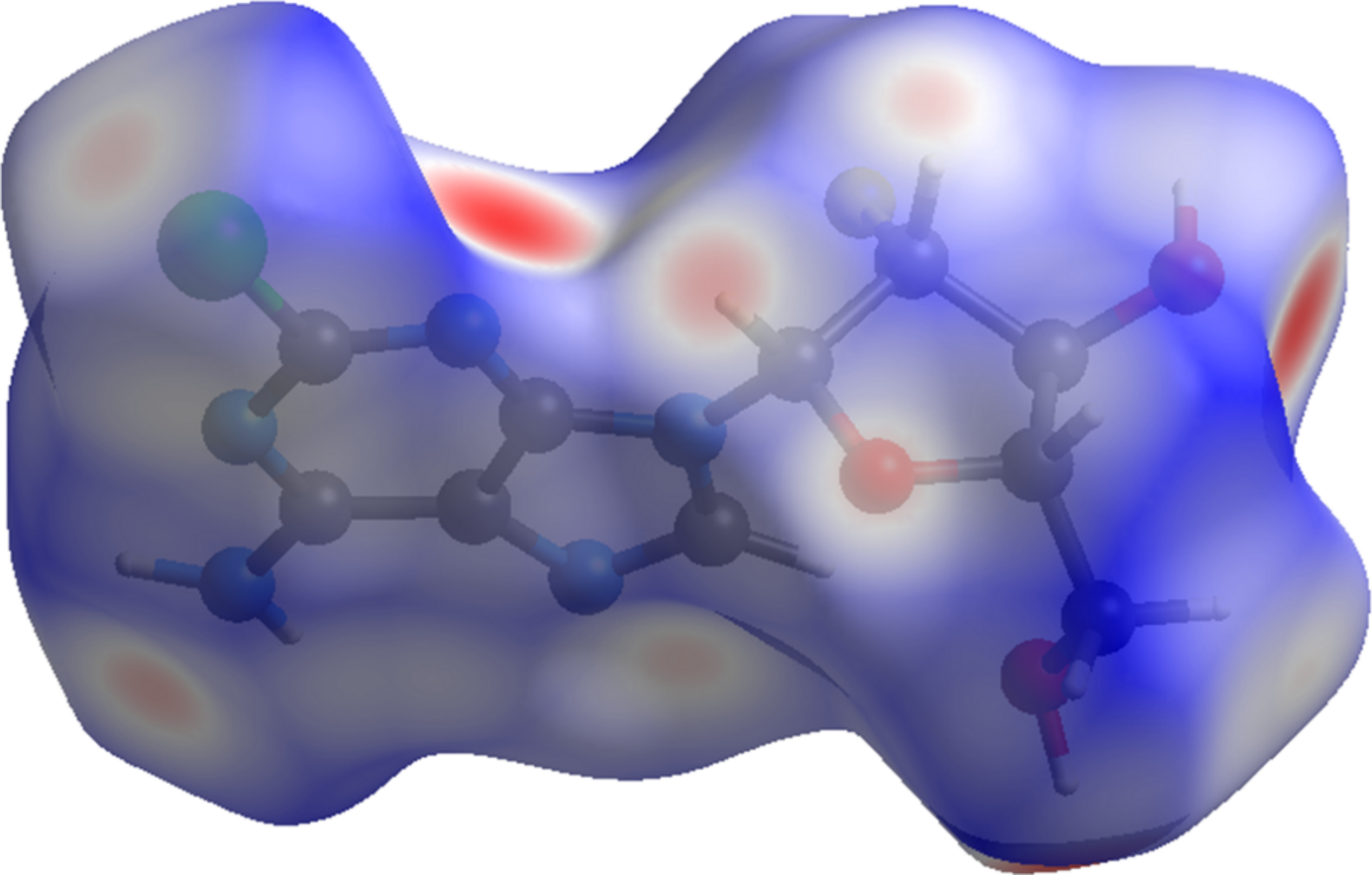
The Hirshfeld surface of clofarabine. Inter­molecular contacts longer than the sums of the van der Waals radii are colored blue, and contacts shorter than the sums of the radii are colored red. Contacts equal to the sums of radii are white.

The volume enclosed by the Hirshfeld surface of clofarabine (Fig. 7 ▸; Hirshfeld, 1977 ▸; Spackman *et al.*, 2021 ▸) is 299.22 Å^3^ or 97.94% of 1/4 of the unit-cell volume. The packing density is thus typical. The only significant close contacts (red in Fig. 9) involve the hydrogen bonds. The volume/non-hydrogen atom is smaller than normal, at 15.3 Å^3^.

The Bravais–Friedel–Donnay–Harker (Bravais, 1866 ▸; Friedel, 1907 ▸; Donnay & Harker, 1937 ▸) algorithm suggests that we might expect elongated morphology for crystallites of clofarabine, with [100] as the long axis. A second-order spherical harmonic model for preferred orientation was included. The texture index was 1.017, indicating that the preferred orientation was slight in this rotated capillary specimen.

## 4. Database survey

A reduced cell search in the Cambridge Structural Database (CSD, 2026.1.0; Groom *et al.*, 2016 ▸), combined with the chemistry C, H, Cl, F, N, and O only, yielded no hits.

## 5. Synthesis and crystallization

Clofarabine is a commercial reagent, purchased from TargetMol (Batch #132343), and was used as-received.

## 6. Refinement

Crystal data, data collection and structure refinement details are summarized in Table 2 ▸. The white powder was packed into a 1.5 mm diameter Kapton capillary, and rotated during the measurement at ∼50 Hz. The powder pattern was measured at 295 K at beam line 11-BM (Lee *et al.*, 2008 ▸; Wang *et al.*, 2008 ▸; Antao *et al.*, 2008 ▸) of the Advanced Photon Source at Argonne National Laboratory using a wavelength of 0.4687342 Å from 0.5–50° 2θ with a step size of 0.001° and a counting time of 0.1 sec step^−1^. The high-resolution powder diffraction data were collected using twelve silicon crystal analyzers that allow for high angular resolution, high precision, and accurate peak positions. A mixture of silicon (NIST SRM 640c) and alumina (NIST SRM 676a) standards (ratio Al_2_O_3_:Si = 2:1 by weight) was used to calibrate the instrument and refine the monochromatic wavelength used in the experiment.

The pattern was indexed on a primitive ortho­rhom­bic unit cell with *a* = 5.06685, *b* = 10.79310, *c* = 22.33892 Å, *V* = 1225.6 Å^3^, and *Z* = 4 using *N-TREOR* as incorporated into *EXPO2014* (Altomare *et al.*, 2013 ▸). The suggested space group was *P*2_1_2_1_2_1_, which was confirmed by the successful solution and refinement of the structure.

The mol­ecular structure of clofarabine was downloaded from PubChem (Kim *et al.*, 2023 ▸) as Conformer3D_COMPOUND_CID_119182.sdf. It was converted to a *.mol2 file using *Mercury* (Macrae *et al.*, 2020 ▸), and to a Fenske–Hall *Z*-matrix using *OpenBabel* (O’Boyle *et al.*, 2011 ▸). The structure was solved using parallel tempering techniques as implemented in *FOX* (Favre-Nicolin & Černý, 2002 ▸).

Rietveld refinement was carried out using *GSAS-II* (Toby & Von Dreele, 2013 ▸). Only the 2.0–35.0° portion of the pattern was included in the refinements (*d*_min_ = 0.779 Å). All non-H bond distances and angles were subjected to restraints, based on a *Mercury* Mogul geometry check (Sykes *et al.*, 2011 ▸; Bruno *et al.*, 2004 ▸). The Mogul average and standard deviation for each qu­antity were used as the restraint parameters. The aromatic fused ring system was restrained to be planar. The restraints contributed 1.4% to the overall *χ^2^*. The hydrogen atoms were included in calculated positions, which were recalculated during the refinement using *Materials Studio* (Dassault Systèmes, 2025 ▸). The Cl atom was refined anisotropically. The other *U*_iso_(H) values were grouped by chemical similarity. The peak profiles were described using the generalized microstrain model (Stephens, 1999 ▸). The background was modeled using a six-term shifted Chebyshev polynomial, with a peak at 5.89° to model the scattering from the Kapton capillary and any amorphous component of the sample.

**Figure 8:**
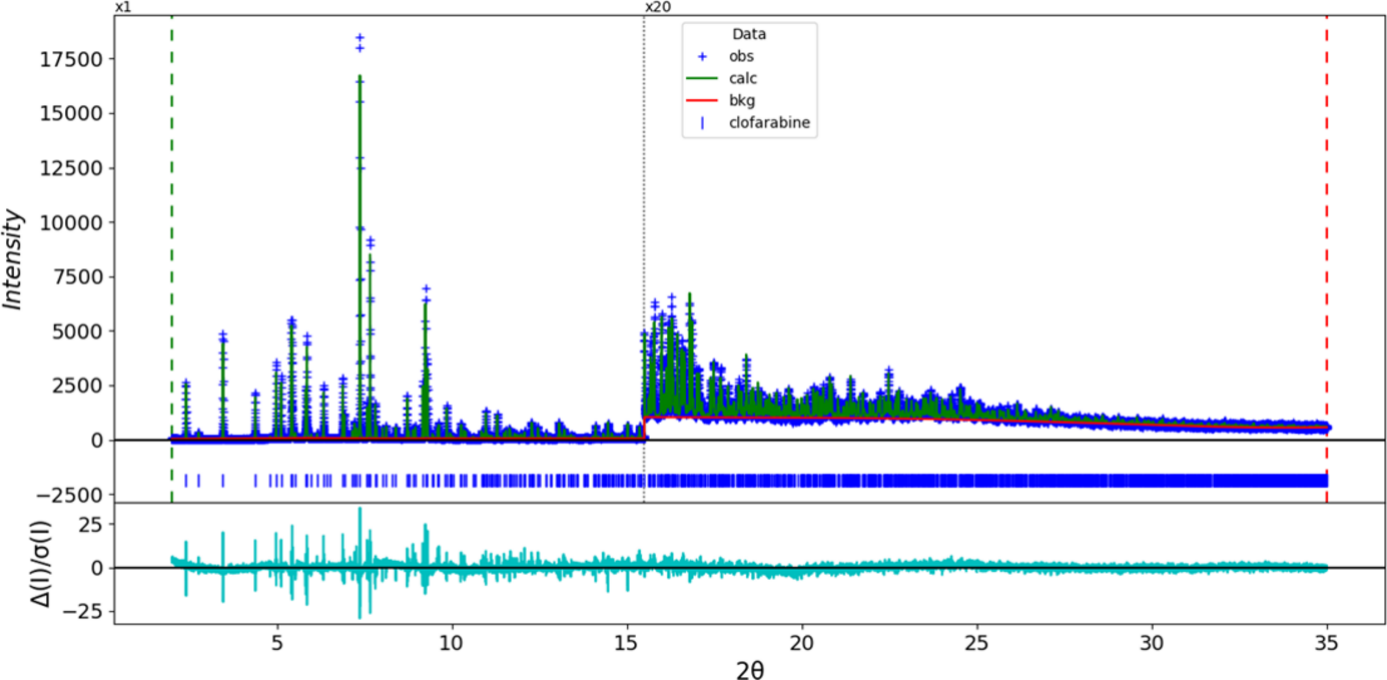
The Rietveld plot for clofarabine. The blue crosses represent the observed data points, and the green line is the calculated pattern. The cyan curve is the normalized error plot, and the red line is the background curve. The blue tick marks indicate the peak positions. The vertical scale has been multiplied by a factor of 20× for 2θ > 15.5°.

The final refinement of 92 variables using 33,001 observations and 56 restraints yielded the residuals *R*_wp_ = 0.0856 and GOF = 2.11. The largest peak (0.65 Å from Cl1) and hole (0.36 Å from Cl1) in the difference-Fourier map are 0.459 (12) and −0.594 (12) *e* Å^−3^, respectively. The final Rietveld plot is shown in Fig. 8 ▸. The largest features in the normalized error plot are in the positions and shapes of some of the strong low-angle peaks, and may indicate a change in the specimen during the measurement.

The crystal structure of clofarabine was optimized (fixed experimental unit cell) with density functional theory techniques using *VASP* (Kresse & Furthmüller, 1996 ▸) through the *MedeA* graphical inter­face (Materials Design, 2024 ▸). The calculation was carried out on 32 cores of a 144-core (768 Gb memory) HPE Superdome Flex 280 Linux server at North Central College. The calculation used the GGA-PBE functional, a plane wave cutoff energy of 400.0 eV, and a *k*-point spacing of 0.5 Å^−1^ leading to a 3 × 2 × 1 mesh, and took ∼8.1 h. Single-point density functional theory calculations (fixed experimental cell) and population analysis were carried out using *CRYSTAL23* (Erba *et al.*, 2023 ▸). Fixed experimental cell) and population analysis were carried out using *CRYSTAL17* (Dovesi *et al.*, 2018 ▸). The basis sets for the H, C, N and O atoms in the calculation were those of Gatti *et al.* (1994 ▸), and those for F and Cl were those of Peintinger *et al.* (2013 ▸). The calculations were run on a 3.5 GHz PC using 8 *k*-points and the B3LYP functional, and took ∼1.4 h.

## Supplementary Material

Crystal structure: contains datablock(s) clofarabine, clofarabine_VASP. DOI: 10.1107/S2056989026006584/hb8221sup1.cif

CCDC references: 2564142, 2564141

Additional supporting information:  crystallographic information; 3D view; checkCIF report

## Figures and Tables

**Table 1:** Hydrogen-bond geometry (Å, °) for clofarabine_VASP[Chem scheme1]

*D*—H⋯*A*	*D*—H	H⋯*A*	*D*⋯*A*	*D*—H⋯*A*
O4—H28⋯N7^i^	1.01	1.72	2.726	173
O5—H29⋯N8^ii^	0.99	1.86	2.831	167
N10—H30⋯O4^iii^	1.03	1.87	2.854	159
N10—H31⋯N10^iv^	1.02	2.38	3.271	145
C11—H21⋯F2^v^	1.10	2.27	3.208	142
C11—H21⋯O5^vi^	1.10	2.60	3.531	142
C12—H22⋯O3^vii^	1.10	2.40	3.396	149
C15—H26⋯Cl1^viii^	1.11	2.77	3.570	129
C17—H27⋯F2^ix^	1.09	2.40	3.177	127

Symmetry codes: (i) 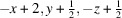; (ii) 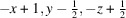; (iii) 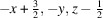; (iv) 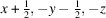; (v) 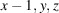; (vi) 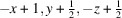; (vii) 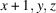; (viii) 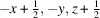; (ix) 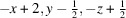.

**Table 2:** Experimental details

	clofarabine
Crystal data	
Chemical formula	C_10_H_11_ClFN_5_O_3_
*M*_r_	303.68
Crystal system, space group	Orthorhombic, *P*2_1_2_1_2_1_
Temperature (K)	295
*a*, *b*, *c* (Å)	5.067481 (12), 10.79402 (2), 22.34124 (5)
*V* (Å^3^)	1222.03 (1)
*Z*	4
Radiation type	Synchrotron, λ = 0.46873 Å
μ (mm^−1^)	0.04
Specimen shape, size (mm)	Cylinder, 2.0 × 1.5

Data collection	
Diffractometer	11-BM, APS
Specimen mounting	Kapton capillary
Data collection mode	Transmission
Scan method	Step
2θ values (°)	2θ_min_ = 0.510, 2θ_max_ = 49.995, 2θ_step_ = 0.001

Refinement	
*R* factors and goodness of fit	*R*_p_ = 0.072, *R*_wp_ = 0.085, *R*_exp_ = 0.041, *R*(*F*^2^) = 0.06753, χ^2^ = 4.439
No. of parameters	92
No. of restraints	56
(Δ/σ)_max_	13.147

Computer programs: *GSAS-II* (Toby & Von Dreele, 2013 ▸) and *DIAMOND* (Brandenburg & Putz, 2025 ▸).

## supplementary crystallographic information

### 2-Chloro-9-(2-deoxy-2-fluoro-β-D-arabinofuranosyl)-9H-purin-6-amine (clofarabine) Crystal data

**Table:**

C_10_H_11_ClFN_5_O_3_	*Z* = 4
*M**_r_* = 303.68	*D*_x_ = 1.651 Mg m^−^^3^
Orthorhombic, *P*2_1_2_1_2_1_	Synchrotron radiation, λ = 0.46873 Å
*a* = 5.067481 (12) Å	µ = 0.04 mm^−^^1^
*b* = 10.79402 (2) Å	*T* = 295 K
*c* = 22.34124 (5) Å	cylinder, 2.0 × 1.5 mm
*V* = 1222.03 (1) Å^3^	

### 2-Chloro-9-(2-deoxy-2-fluoro-β-D-arabinofuranosyl)-9H-purin-6-amine (clofarabine) Data collection

**Table:**

11-BM, APS diffractometer	Scan method: step
Specimen mounting: Kapton capillary	2θ_min_ = 0.510°, 2θ_max_ = 49.995°, 2θ_step_ = 0.001°
Data collection mode: transmission	

### 2-Chloro-9-(2-deoxy-2-fluoro-β-D-arabinofuranosyl)-9H-purin-6-amine (clofarabine) Refinement

**Table:**

Least-squares matrix: full	92 parameters
*R*_p_ = 0.072	56 restraints
*R*_wp_ = 0.085	16 constraints
*R*_exp_ = 0.041	Weighting scheme based on measured s.u.'s
*R*(*F*^2^) = 0.06753	(Δ/σ)_max_ = 13.147
49486 data points	Background function: Background function: "chebyschev-1" function with 6 terms: 42.46(6), -8.82(9), -9.57(8), 2.83(8), -1.00(7), 2.56(7), Background peak parameters: pos, int, sig, gam: 5.889(10), 4.22(8)e3, 4.12(15)e3, 0.100,
Profile function: Finger-Cox-Jephcoat function parameters U, V, W, X, Y, SH/L: peak variance(Gauss) = Utan(Th)^2^+Vtan(Th)+W: peak HW(Lorentz) = X/cos(Th)+Ytan(Th); SH/L = S/L+H/L U, V, W in (centideg)^2^, X & Y in centideg 1.163, -0.126, 0.063, 0.000, 0.000, 0.002,	Preferred orientation correction: Simple spherical harmonic correction Order = 2 Coefficients: 0:0:C(2,0) = -0.2134(29); 0:0:C(2,2) = 0.199(4)

### 2-Chloro-9-(2-deoxy-2-fluoro-β-D-arabinofuranosyl)-9H-purin-6-amine (clofarabine) Fractional atomic coordinates and isotropic or equivalent isotropic displacement parameters (Å^2^)

**Table:**

	*x*	*y*	*z*	*U*_iso_*/*U*_eq_	
Cl1	0.0189 (2)	0.16598 (9)	0.02919 (5)	0.0491	
F2	0.8827 (3)	0.21882 (16)	0.24051 (9)	0.0404 (4)*	
O3	0.3887 (4)	0.0218 (2)	0.29218 (10)	0.0404 (4)*	
O4	0.8336 (4)	0.1997 (2)	0.38062 (10)	0.0404 (4)*	
O5	0.7624 (5)	−0.1464 (2)	0.33800 (11)	0.0543 (8)*	
N6	0.5950 (5)	0.0280 (2)	0.19828 (9)	0.0276 (3)*	
N7	0.8275 (5)	−0.1145 (2)	0.14655 (11)	0.0276 (3)*	
N8	0.3110 (5)	0.1027 (2)	0.11892 (10)	0.0276 (3)*	
N9	0.3786 (5)	−0.0050 (2)	0.02505 (9)	0.0276 (3)*	
N10	0.7125 (5)	−0.1486 (2)	0.01227 (11)	0.0276 (3)*	
C11	0.4845 (5)	0.1019 (2)	0.24741 (11)	0.0404 (4)*	
C12	0.7673 (5)	0.1167 (3)	0.33294 (12)	0.0404 (4)*	
C13	0.6750 (5)	0.1895 (2)	0.27873 (12)	0.0404 (4)*	
C14	0.5214 (6)	0.0417 (3)	0.34917 (13)	0.0404 (4)*	
C15	0.5802 (7)	−0.0816 (3)	0.37589 (16)	0.0543 (8)*	
C16	0.5031 (5)	0.0297 (3)	0.14087 (9)	0.0276 (3)*	
C17	0.7914 (6)	−0.0585 (3)	0.19924 (11)	0.0276 (3)*	
C18	0.6444 (6)	−0.0584 (3)	0.11009 (10)	0.0276 (3)*	
C19	0.5783 (6)	−0.0731 (3)	0.04870 (10)	0.0276 (3)*	
C20	0.2718 (6)	0.0791 (3)	0.06104 (11)	0.0276 (3)*	
H21	0.30900	0.14393	0.23107	0.0526*	
H22	0.93174	0.06037	0.32382	0.0526*	
H23	0.57838	0.27853	0.28915	0.0526*	
H24	0.38326	0.09796	0.37362	0.0526*	
H25	0.40893	−0.13251	0.38656	0.0706*	
H26	0.68813	−0.06089	0.41746	0.0706*	
H27	0.89907	−0.08325	0.23913	0.0358*	
H28	0.97473	0.25980	0.36946	0.0526*	
H29	0.75868	−0.23048	0.35566	0.0706*	
H30	0.66866	−0.15293	−0.03273	0.0358*	
H31	0.82978	−0.21654	0.02850	0.0358*	

### 2-Chloro-9-(2-deoxy-2-fluoro-β-D-arabinofuranosyl)-9H-purin-6-amine (clofarabine) Atomic displacement parameters (Å^2^)

**Table:**

	*U*^11^	*U*^22^	*U*^33^	*U*^12^	*U*^13^	*U*^23^
Cl1	0.0463 (9)	0.0535 (9)	0.0474 (10)	0.0085 (15)	−0.0268 (15)	0.0218 (16)

### 2-Chloro-9-(2-deoxy-2-fluoro-β-D-arabinofuranosyl)-9H-purin-6-amine (clofarabine) Geometric parameters (Å, º)

**Table:**

Cl1—C20	1.740 (2)	C14—C12	1.529 (3)
F2—C13	1.392 (2)	C14—C15	1.489 (3)
O3—C11	1.408 (3)	C14—H24	1.076 (3)
O3—C14	1.456 (3)	C15—O5	1.435 (4)
O4—C12	1.432 (3)	C15—C14	1.489 (3)
O4—H28	0.997 (2)	C15—H25	1.055 (4)
O5—C15	1.435 (4)	C15—H26	1.101 (4)
O5—H29	0.990 (2)	C16—N6	1.365 (2)
N6—C11	1.468 (3)	C16—N8	1.345 (2)
N6—C16	1.365 (2)	C16—C18	1.375 (2)
N6—C17	1.364 (2)	C17—N6	1.364 (2)
N7—C17	1.336 (3)	C17—N7	1.336 (3)
N7—C18	1.375 (2)	C17—H27	1.079 (2)
N8—C16	1.345 (2)	C18—N7	1.375 (2)
N8—C20	1.333 (2)	C18—C16	1.375 (2)
N9—C19	1.357 (2)	C18—C19	1.421 (3)
N9—C20	1.328 (2)	C19—N9	1.357 (2)
N10—C19	1.338 (3)	C19—N10	1.338 (3)
N10—H30	1.031 (2)	C19—C18	1.421 (3)
N10—H31	1.011 (2)	C20—Cl1	1.740 (2)
C11—O3	1.408 (3)	C20—N8	1.333 (2)
C11—N6	1.468 (3)	C20—N9	1.328 (2)
C11—C13	1.522 (2)	H21—C11	1.064 (3)
C11—H21	1.064 (3)	H22—C12	1.052 (3)
C12—O4	1.432 (3)	H23—C13	1.103 (3)
C12—C13	1.518 (2)	H24—C14	1.076 (3)
C12—C14	1.529 (3)	H25—C15	1.055 (4)
C12—H22	1.052 (3)	H26—C15	1.101 (4)
C13—F2	1.392 (2)	H27—C17	1.079 (2)
C13—C11	1.522 (2)	H28—O4	0.997 (2)
C13—C12	1.518 (2)	H29—O5	0.990 (2)
C13—H23	1.103 (3)	H30—N10	1.031 (2)
C14—O3	1.456 (3)	H31—N10	1.011 (2)
			
C11—O3—C14	111.81 (18)	C12—C13—H23	114.8 (2)
C12—O4—H28	112.9 (2)	O3—C14—C12	104.3 (2)
C15—O5—H29	101.5 (3)	O3—C14—C15	108.1 (3)
C11—N6—C16	124.40 (19)	C12—C14—C15	113.9 (3)
C11—N6—C17	129.7 (2)	O3—C14—H24	103.1 (2)
C16—N6—C17	105.86 (14)	C12—C14—H24	110.6 (3)
C17—N7—C18	103.30 (17)	C15—C14—H24	115.5 (3)
C16—N8—C20	110.43 (17)	O5—C15—C14	109.2 (3)
C19—N9—C20	115.96 (16)	O5—C15—H25	114.1 (3)
C19—N10—H30	120.8 (2)	C14—C15—H25	113.1 (3)
C19—N10—H31	121.5 (2)	O5—C15—H26	106.1 (3)
H30—N10—H31	116.3 (2)	C14—C15—H26	104.9 (3)
O3—C11—N6	109.2 (2)	H25—C15—H26	108.9 (3)
O3—C11—C13	105.89 (15)	N6—C16—N8	126.72 (15)
N6—C11—C13	116.1 (2)	N6—C16—C18	106.42 (13)
O3—C11—H21	102.6 (3)	N8—C16—C18	126.86 (15)
N6—C11—H21	107.12 (19)	N6—C17—N7	113.30 (17)
C13—C11—H21	115.0 (2)	N6—C17—H27	123.5 (2)
O4—C12—C13	110.0 (2)	N7—C17—H27	123.1 (3)
O4—C12—C14	110.2 (3)	N7—C18—C16	111.09 (15)
C13—C12—C14	102.26 (17)	N7—C18—C19	132.98 (18)
O4—C12—H22	108.7 (2)	C16—C18—C19	115.92 (15)
C13—C12—H22	112.9 (3)	N9—C19—N10	118.15 (19)
C14—C12—H22	112.7 (3)	N9—C19—C18	119.43 (17)
F2—C13—C11	109.8 (2)	N10—C19—C18	122.37 (19)
F2—C13—C12	112.0 (2)	Cl1—C20—N8	113.75 (17)
C11—C13—C12	103.89 (15)	Cl1—C20—N9	114.91 (16)
F2—C13—H23	105.51 (19)	N8—C20—N9	131.13 (19)
C11—C13—H23	110.9 (2)		

### (clofarabine_VASP) Crystal data

**Table:**

C_10_H_11_ClFN_5_O_3_	*c* = 22.33879 Å
*M**_r_* = 360.68	*V* = 1221.61 Å^3^
Orthorhombic, *P*2_1_2_1_2_1_	*Z* = 4
*a* = 5.06687 Å	*D*_x_ = 1.651 Mg m^−^^3^
*b* = 10.79280 Å	

### (clofarabine_VASP) Data collection

**Table:**

*h* = →	*l* = →
*k* = →	

### (clofarabine_VASP) Fractional atomic coordinates and isotropic or equivalent isotropic displacement parameters (Å^2^)

**Table:**

	*x*	*y*	*z*	*B*_iso_*/*B*_eq_	
Cl1	0.01212	0.16968	0.02898		
F2	0.90535	0.21606	0.23899		
O3	0.38561	0.02587	0.29060		
O4	0.84115	0.19497	0.38208		
O5	0.75419	−0.14849	0.34117		
N6	0.59832	0.02849	0.19629		
N7	0.81396	−0.11960	0.14513		
N8	0.31324	0.10597	0.11693		
N9	0.37245	−0.00306	0.02375		
N10	0.68616	−0.15566	0.00827		
C11	0.49010	0.10293	0.24534		
C12	0.77245	0.11804	0.33283		
C13	0.69128	0.18783	0.27665		
C14	0.52255	0.04396	0.34742		
C15	0.57085	−0.08032	0.37626		
C16	0.50115	0.03082	0.13866		
C17	0.78576	−0.06389	0.19766		
C18	0.63647	−0.06134	0.10719		
C19	0.56958	−0.07502	0.04585		
C20	0.26192	0.07985	0.05972		
H21	0.33140	0.15735	0.22472		
H22	0.93523	0.05413	0.32150		
H23	0.59644	0.27638	0.28803		
H24	0.39549	0.09995	0.37712		
H25	0.37979	−0.12899	0.38061		
H26	0.64633	−0.06322	0.42197		
H27	0.88790	−0.08940	0.23872		
H28	0.97473	0.25980	0.36946		
H29	0.75849	−0.23464	0.35654		
H30	0.65698	−0.14890	−0.03741		
H31	0.85246	−0.20128	0.02116		

### (clofarabine_VASP) Hydrogen-bond geometry (Å, º)

**Table:**

*D*—H···*A*	*D*—H	H···*A*	*D*···*A*	*D*—H···*A*
O4—H28···N7^i^	1.01	1.72	2.726	173
O5—H29···N8^ii^	0.99	1.86	2.831	167
N10—H30···O4^iii^	1.03	1.87	2.854	159
N10—H31···N10^iv^	1.02	2.38	3.271	145
C11—H21···F2^v^	1.10	2.27	3.208	142
C11—H21···O5^vi^	1.10	2.60	3.531	142
C12—H22···O3^vii^	1.10	2.40	3.396	149
C15—H26···Cl1^viii^	1.11	2.77	3.570	129
C17—H27···F2^ix^	1.09	2.40	3.177	127

Symmetry codes: (i) −*x*+2, *y*+1/2, −*z*+1/2; (ii) −*x*+1, *y*−1/2, −*z*+1/2; (iii) −*x*+3/2, −*y*, *z*−1/2; (iv) *x*+1/2, −*y*−1/2, −*z*; (v) *x*−1, *y*, *z*; (vi) −*x*+1, *y*+1/2, −*z*+1/2; (vii) *x*+1, *y*, *z*; (viii) −*x*+1/2, −*y*, *z*+1/2; (ix) −*x*+2, *y*−1/2, −*z*+1/2.

## References

[bb1] Altomare, A., Cuocci, C., Giacovazzo, C., Moliterni, A., Rizzi, R., Corriero, N. & Falcicchio, A. (2013). *J. Appl. Cryst.* **46**, 1231–1235.

[bb2] Antao, S. M., Hassan, I., Wang, J., Lee, P. L. & Toby, B. H. (2008). *Can. Mineral.* **46**, 1501–1509.

[bb3] Bernstein, J., Davis, R. E., Shimoni, L. & Chang, N. L. (1995). *Angew. Chem. Int. Ed. Eng* **34**, 1555–1573.

[bb4] Bonate, P. L., Arthaud, L., Cantrell, W. R., Stephenson, K., Secrist, J. A. & Weitman, S. (2006). *Nat. Rev. Drug Discov.* **5**, 855–863. 10.1038/nrd2055 17016426

[bb5] Brandenburg, K. & Putz, H. (2025). *DIAMOND V 5.1.1*. Crystal Impact, Bonn, Germany.

[bb6] Bravais, A. (1866). *Etudes Cristallographiques.* Paris: Gauthier Villars.

[bb7] Bruno, I. J., Cole, J. C., Kessler, M., Luo, J., Motherwell, W. D. S., Purkis, L. H., Smith, B. R., Taylor, R., Cooper, R. I., Harris, S. E. & Orpen, A. G. (2004). *J. Chem. Inf. Comput. Sci.* **44**, 2133–2144. 10.1021/ci049780b 15554684

[bb8] Dassault Systèmes (2025). *BIOVIA Materials Studio 2026*. BIOVIA, San Diego, USA.

[bb9] Donnay, J. D. H. & Harker, D. (1937). *Am. Mineral.* **22**, 446–467.

[bb41] Dovesi, R., Erba, A., Orlando, R., Zicovich-Wilson, C. M., Civalleri, B., Maschio, L., Rérat, M., Casassa, S., Baima, J., Salustro, S. & Ki

[bb10] Erba, A., Desmarais, J. K., Casassa, S., Civalleri, B., Donà, L., Bush, I. J., Searle, B., Maschio, L., Daga, L.-E., Cossard, A., Ribaldone, C., Ascrizzi, E., Marana, N. L., Flament, J.-P. & Kirtman, B. (2023). *J. Chem. Theory Comput.* **19**, 6891–6932. 10.1021/acs.jctc.2c00958 PMC10601489 36502394

[bb11] Etter, M. C. (1990). *Acc. Chem. Res.* **23**, 120–126.

[bb12] Favre-Nicolin, V. & Černý, R. (2002). *J. Appl. Cryst.* **35**, 734–743.

[bb13] Friedel, G. (1907). *Bull. Soc. Française Minéral.* **30**, 326–455.

[bb14] Gatti, C., Saunders, V. R. & Roetti, C. (1994). *J. Chem. Phys.* **101**, 10686–10696.

[bb15] Groom, C. R., Bruno, I. J., Lightfoot, M. P. & Ward, S. C. (2016). *Acta Cryst.* B**72**, 171–179. 10.1107/S2052520616003954 PMC4822653 27048719

[bb16] Hirshfeld, F. L. (1977). *Theoret. Chem. Acta* **44**, 129-138.

[bb17] Kabekkodu, S., Dosen, A. & Blanton, T. N. (2024). *Powder Diffr.* **39**, 47–59.

[bb19] Kaduk, J. A., Crowder, C. E., Zhong, K., Fawcett, T. G. & Suchomel, M. R. (2014). *Powder Diffr.* **29**, 269–273.

[bb20] Kim, S., Chen, J., Cheng, T., Gindulyte, A., He, J., He, S., Li, Q., Shoemaker, B. A., Thiessen, P. A., Yu, B., Zaslavsky, L., Zhang, J. & Bolton, E. E. (2023). *Nucleic Acids Res.* **51**, D1373–D1380. 10.1093/nar/gkac956 PMC9825602 36305812

[bb21] Kresse, G. & Furthmüller, J. (1996). *Comput. Mat. Sci.* **6**, 15–50.

[bb22] Lee, P. L., Shu, D., Ramanathan, M., Preissner, C., Wang, J., Beno, M. A., Von Dreele, R. B., Ribaud, L., Kurtz, C., Antao, S. M., Jiao, X. & Toby, B. H. (2008). *J. Synch. Rad.* **15**, 427–432. 10.1107/S0909049508018438 18728312

[bb23] Macrae, C. F., Sovago, I., Cottrell, S. J., Galek, P. T. A., McCabe, P., Pidcock, E., Platings, M., Shields, G. P., Stevens, J. S., Towler, M. & Wood, P. A. (2020). *J. Appl. Cryst.* **53**, 226–235. 10.1107/S1600576719014092 PMC6998782 32047413

[bb24] Materials Design. (2024). *MedeA 3.7.2*. Materials Design Inc., San Diego, USA.

[bb25] MDI (2026). *JADE Pro version 9.5*. Materials Data, Livermore, USA.

[bb26] Montgomery, J. A. & Secrist, J. A. (1991). United States Patent 5,034,518.

[bb27] Montgomery, J. A. & Secrist, J. A. (1997). United States Patent 5,661,136.

[bb28] Motherwell, W. D. S., Shields, G. P. & Allen, F. H. (2000). *Acta Cryst.* B**56**, 857–871. 10.1107/S0108768100007230 11006562

[bb29] O’Boyle, N. M., Banck, M., James, C. A., Morley, C., Vandermeersch, T. & Hutchison, G. R. (2011). *J. Chem. Informatics* **3**, 33. 10.1186/1758-2946-3-33 PMC3198950 21982300

[bb30] Peintinger, M. F., Vilela Oliveira, D. & Bredow, T. (2013). *J. Comput. Chem.* **34**, 451–459. 10.1002/jcc.23153 23115105

[bb31] Silk Scientific. (2013). *UN-SCAN-IT 7.0*. Silk Scientific Corporation, Orem, USA.

[bb32] Spackman, P. R., Turner, M. J., McKinnon, J. J., Wolff, S. K., Grimwood, D. J., Jayatilaka, D. & Spackman, M. A. (2021). *J. Appl. Cryst.* **54**, 1006–1011. 10.1107/S1600576721002910 PMC8202033 34188619

[bb33] Stephens, P. W. (1999). *J. Appl. Cryst.* **32**, 281–289.

[bb36] Streek, J. van de & Neumann, M. A. (2014). *Acta Cryst.* B**70**, 1020–1032. 10.1107/S2052520614022902 PMC4468513 25449625

[bb34] Sykes, R. A., McCabe, P., Allen, F. H., Battle, G. M., Bruno, I. J. & Wood, P. A. (2011). *J. Appl. Cryst.* **44**, 882–886. 10.1107/S0021889811014622 PMC3246811 22477784

[bb35] Toby, B. H. & Von Dreele, R. B. (2013). *J. Appl. Cryst.* **46**, 544–549.

[bb37] Wang, J., Toby, B. H., Lee, P. L., Ribaud, L., Antao, S. M., Kurtz, C., Ramanathan, M., Von Dreele, R. B. & Beno, M. A. (2008). *Rev. Sci. Instr.* **79**, 085105. 10.1063/1.2969260 19044378

[bb38] Wavefunction (2025). *Spartan ’24. V. 1.3.1.* Wavefunction Inc., Irvine, USA.

[bb39] Wheatley, A. M. & Kaduk, J. A. (2019). *Powder Diffr.* **34**, 35–43.

[bb40] Xia, C., Zhang, X., Deng, Y., Miao, W. & Tao, Y. (2011). Chinese Patent CN101497640A.

